# Anti-Inflammatory Effects, SAR, and Action Mechanism of Monoterpenoids from *Radix* *Paeoniae Alba* on LPS-Stimulated RAW 264.7 Cells

**DOI:** 10.3390/molecules22050715

**Published:** 2017-04-29

**Authors:** Xiaoxu Bi, Li Han, Tiange Qu, Yu Mu, Peipei Guan, Xiaodan Qu, Zhanyou Wang, Xueshi Huang

**Affiliations:** 1 College of Life and Health Sciences, Northeastern University, Shenyang 110819, China; xiaoxu.bi@hotmail.com (X.B.); muyu@mail.neu.edu.cn (Y.M.); guanpp@mail.neu.edu.cn (P.G.); xiaodanqu@hotmail.com (X.Q.); wangzy@mail.neu.edu.cn (Z.W.); 2 Dongzhimen Hospital of the First Clinical Medical College, Beijing University of Chinese Medicine, Beijing 100700, China; tiangequ1119@126.com

**Keywords:** *Radix Paeoniae Alba*, monoterpenoids, paeoniflorins, paeonidanins, albiflorin, anti-inflammation, SAR, action mechanism

## Abstract

Nine monoterpenoids from *Radix Paeoniae Alba*, including paeoniflorin derivatives, paeoniflorin (PF), 4-*O*-methylpaeoniflorin (MPF), 4-*O*-methylbenzoylpaeoniflorin (MBPF); paeonidanin derivatives, paeonidanin (PD), paeonidanin A (PDA), albiflorin derivatives, albiflorin (AF), benzoylalbiflorin (BAF), galloylalbiflorin (GAF), and debenzoylalbiflorin (DAF), were obtained in our previous phytochemistry investigations. Their anti-inflammatory effects were determined in the present study. The expression and production of pro-inflammatory cytokines in lipopolysaccharides (LPS)-stimulated RAW 264.7 cells were measured using an Elisa assay and nitric oxide (NO) release was determined using the Griess method. The results demonstrated that the most of the monoterpenoids suppressed the LPS-induced production of NO, interleukin-6 (IL-6), and tumor necrosis factor alpha (TNF-α). The anti-inflammatory activities of these monoterpenoids were closely related to their structural characteristics. Paeoniflorins and paeonidanins presented stronger anti-inflammatory activities than those of albiflorin derivatives. Furthermore, the action mechanisms of MBPF, having a strong anti-inflammatory effect, were investigated using quantitative reverse transcription polymerase chain reaction (RT-PCR) and Western blot methods. The results indicated that MBPF could down-regulate the mRNA and protein expression level of inducible nitric oxide synthase (iNOS) in LPS-stimulated RAW 264.7 cells. The mitogen-activated protein kinase (MAPK), phosphatidylinositol 3-kinase (PI3K)/AKT and nuclear factor κB (NF-κB) signaling pathways are involved in mediating the role of MBPF in suppressing the expression and production of pro-inflammatory cytokines in RAW 264.7 cells.

## 1. Introduction

*Radix Paeoniae Alba*, the dried rhizome of *Paeonia lactiflora* Pall., is a traditional Chinese medicine that has been used in a number of Asian countries for treatment of rheumatoid arthritis, to alleviate inflammation, amenorrhea, epistaxis, abdominal pain, and other symptoms [1,2,3]. Monoterpenes and monoterpene glycosides are the primary chemical constituents of the roots of *P. lactiflora* [4,5,6]. In our previous phytochemical investigations, nine monoterpenes, paeoniflorin (PF), 4-*O*-methylpaeoniflorin (MPF), albiflorin (AF), paeonidanin (PD), 4-*O*-methylbenzoylpaeoniflorin (MBPF), benzoylalbiflorin (BAF), paeonidanin A (PDA), galloylalbiflorin (GAF), debenzoylalbiflorin (DAF), were isolated from *P. lactiflora* [7]. PF and AF derivatives had been proven to possess anti-inflammatory potential [4,8,9] and can suppress interleukin signaling and the Toll receptor signaling pathway in lipopolysaccharide (LPS)-induced RAW 264.7 cells [10]. In addition, the literature reported that PF can inhibit the nuclear translocation of nuclear factor κB (NF-κB) to suppress the production of proinflammatory cytokines in LPS-induced RAW 264.7 cells [11].

Macrophages play a crucial role during inflammation, and LPS-stimulated RAW 264.7 cells are widely used for evaluating the anti-inflammatory effects of natural products [12]. LPS binds the Toll-like receptor and activates a set of intracellular signaling cascades, such as mitogen-activated protein kinase (MAPK), phosphatidylinositol 3-kinase (PI3K)/AKT and the NF-κB signal pathways, to induce the expression of cytokines, including factor-α (TNF-α), interleukin-1β (IL-1β), and interleukin-6 (IL-6), and also of inflammatory enzymes, such as inducible nitric oxide synthase (iNOS) [13,14,15,16]. In the present study, comparative anti-inflammatory assays of nine isolated monoterpenoids were carried out, and the structure–activity relationships (SAR) were analyzed. Additionally, the effects of monoterpenoids on Toll receptor signaling were investigated.

## 2. Results and Discussion

### 2.1. SAR and Effect of Monoterpenoids on Anti-Inflammation In Vitro

**Figure 1:**
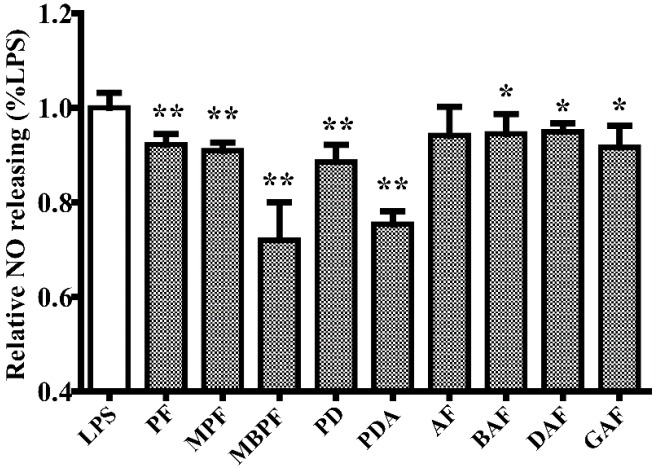
Effect of monoterpenoids (PF, MPF, MBPF, PD, PDA, AF, BAF, DAF, and GAF) on the production of nitric oxide (NO) in lipopolysaccharide (LPS)-induced RAW 264.7 cells. The cells were treated, respectively, with the 33 μM of the nine different compounds for 2 h, and then stimulated with LPS (1 μg/mL) for 24 h. The culture medium was collected to detect the concentrations of nitrite and calculate the relative NO release rate. (PF, paeoniflorin; MPF, 4-*O*-methylpaeoniflorin; MBPF, 4-*O*-methylbenzoylpaeoniflorin; PD, paeonidanin; PDA, paeonidanin A; AF, albiflorin; BAF, benzoylalbiflorin; DAF, debenzoylalbiflorin; GAF, galloylalbiflorin; * *p* < 0.05, ** *p* < 0.01, relative to the LPS group).

To investigate the anti-inflammatory activities of monoterpenoids (PF, MPF, AF, PD, MBPF, BAF, PDA, GAF, and DAF) in vitro, we measured the production of NO, IL-6, and TNF-α in LPS-stimulated RAW 264.7 cells, after treatment with PF, MPF, AF, PD, MBPF, BAF, PDA, GAF, and DAF, respectively. The results showed that paeoniflorin derivatives (PF, MPF, and MBPF) and paeonidanins (PD and PDA) clearly inhibited the production of NO; albiflorin derivatives (BAF, GAF, and DAF) presented weak inhibition of NO release; and albiflorin had no obvious suppression effect at the tested concentration (Figure 1).

**Figure 2:**
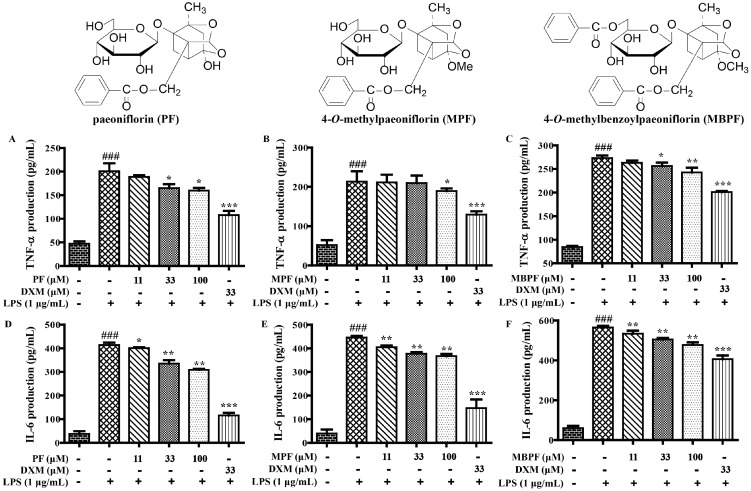
The effects of PF, MPF and MBPF on the production of tumor necrosis factor alpha (TNF-α) and interleukin-6 (IL-6) in LPS-induced RAW 264.7 cells. The cells were treated with different concentrations of PF, MPF and MBPF (0, 11, 33, and 100 μM) for 2 h, and were then stimulated with or without LPS (1 μg/mL) for 12 h. The culture medium was collected to detect the production of TNF-α (**A**–**C**) and IL-6 (**D**–**F**) were measured using Elisa kits. (^###^ *p* < 0.001 vs. control group; * *p* < 0.05, ** *p* < 0.01, *** *p* < 0.001, vs. LPS group).

**Figure 3:**
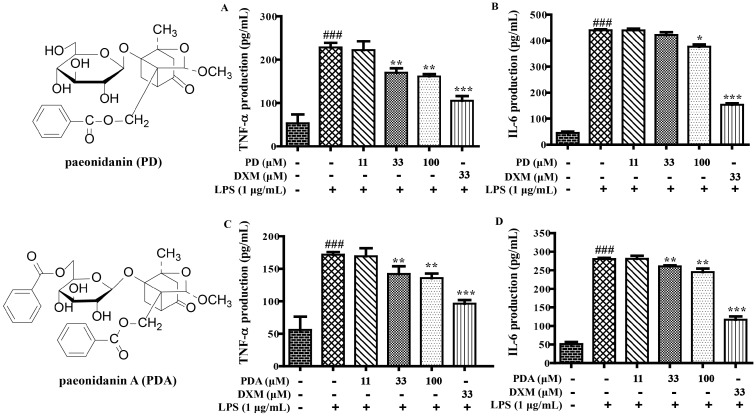
The effects of paeonidanin (PD) and paeonidanin A (PDA) on the production of TNF-α and IL-6 in LPS-induced RAW 264.7 cells. The cells were treated with different concentrations of PD and PDA (0, 11, 33, and 100 μM) for 2 h, and were then stimulated with or without LPS (1 μg/mL) for 12 h. The culture medium was collected to detect the production of TNF-α ((**A**) and (**C**)) and IL-6 ((**B**) and (**D**)) were measured using Elisa kits. (^###^ *p* < 0.001 vs. control group; * *p* < 0.05, ** *p* < 0.01, *** *p* < 0.001, vs. LPS group)

**Figure 4:**
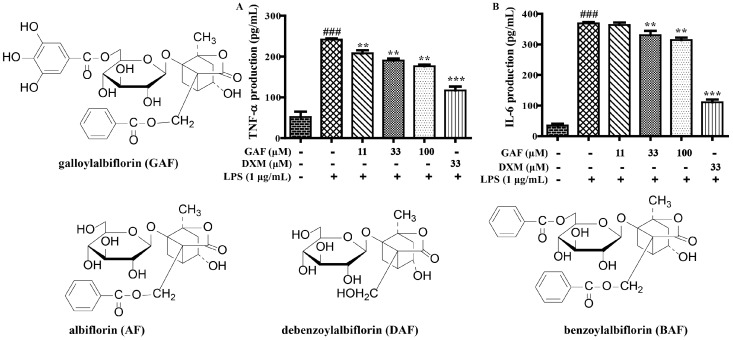
The effect of GAF on production of TNF-α and IL-6 in LPS-induced RAW 264.7 cells. The cells were treated with different concentrations of GAF (0, 11, 33, and 100 μM) for 2 h, and were then stimulated with or without LPS (1 μg/mL) for 12 h. The culture medium was collected to detect the production of TNF-α (**A**) and IL-6 (**B**), which were measured using Elisa kits. (^###^ *p* < 0.01 vs*.* control group; ** *p* < 0.01, *** *p* < 0.001, vs. LPS group). AF, BAF and DAF presented no obvious activities at the tested concentrations.

In addition, the secretion levels of TNF-α and IL-6 were obviously inhibited by paeoniflorins (PF, MPF, and MBPF) and paeonidanins (PD and PDA) in a dose-dependent manner in LPS-stimulated RAW 264.7 cells (Figure 2 and Figure 3). Among the albiflorin derivatives, only GAF significantly suppressed the production of TNF-α and IL-6, and AF, BAF and DAF did not present obvious activities at the tested concentrations (Figure 4). On the other hand, exclusion of the anti-inflammatory activities was due to their cytotoxic effects; the cytotoxicity of the nine monoterpenoids (100 μM) were determined using the 3-(4,5-dimethyl-2-thiazolyl)-2,5-diphenyl-2-*H*-tetrazolium bromide (MTT) assay. The results showed that all of the compounds had no obvious cytotoxicity on RAW 264.7 cells at 100 μM.

All these results further proved that most of the monoterpenoids had anti-inflammatory effects in vitro. The anti-inflammatory activities of these monoterpenoids were closely related to their structure characteristics. Monoterpenoids with structural characteristics of paeoniflorins and paeonidanins presented stronger anti-inflammatory effects compared with those of albiflorin derivatives (PF, MPF, MBPF, PD and PDA vs. AF, BAF, and DAF). The galloyl group, at the sugar moiety of albiflorins, were obviously able to increase the anti-inflammatory effects (GAF vs. AF, BAF, DAF). Among them, MBPF showed a stronger inhibition on the production of NO, IL-6, and TNF-α. Therefore, we further investigated the effects of MBPF on Toll receptor signaling to explore the action mechanism of anti-inflammation.

### 2.2. MBPF Inhibits Expression of iNOS in LPS-Induced RAW 264.7 Cells

**Figure 5:**
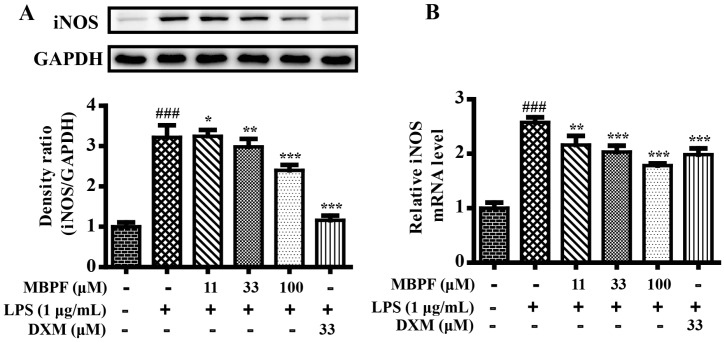
The effects of 4-*O*-methylbenzoylpaeoniflorin (MBPF) on mRNA and protein level expressions of inducible nitric oxide synthase (iNOS) in LPS-induced RAW 264.7 cells. (**A**) Cells were pretreated with increasing concentrations of MBPF (0, 11, 33, and 100 μM) for 2 h and were then stimulated with or without LPS (1 μg/mL) for 24 h. Total proteins of RAW 264.7 cells were prepared for iNOS protein analysis using Western blot; (**B**) cells were pretreated with MBPF (0, 11, 33, and 100 μM) for 2 h and were then stimulated with or without LPS (1 μg/mL) for 6 h. The mRNA levels of iNOS were determined using quantitative real-time Polymerase Chain Reaction (PCR) (**C**). Data represent the mean ± SE from three separate experiments. (^###^ *p* < 0.001 vs*.* control group; * *p* < 0.05, ** *p* < 0.01, *** *p* < 0.001 vs*.* LPS group).

Inducible nitric oxide synthase (iNOS), which catalyzes the production of nitric oxide, plays a significant role in inducing inflammation. Quantitative real-time polymerase chain reaction (RT-qPCR) was performed to determine the effects of MBPF on mRNA expression of iNOS in LPS-induced RAW 264.7 cells. As shown in Figure 5B, MBPF greatly inhibited the mRNA expression levels of iNOS in a concentration-dependent manner in LPS-stimulated RAW 264.7 cells. Western blot analysis confirmed that MBPF inhibited the protein expression level of iNOS (Figure 5A). The results further proved that MBPF had anti-inflammatory effects in vitro.

### 2.3. MBPF Inhibits the Activation of MAPK, PI3K/Akt and NF-κB Signaling Pathway in LPS-Induced RAW 264.7 Cells

**Figure 6:**
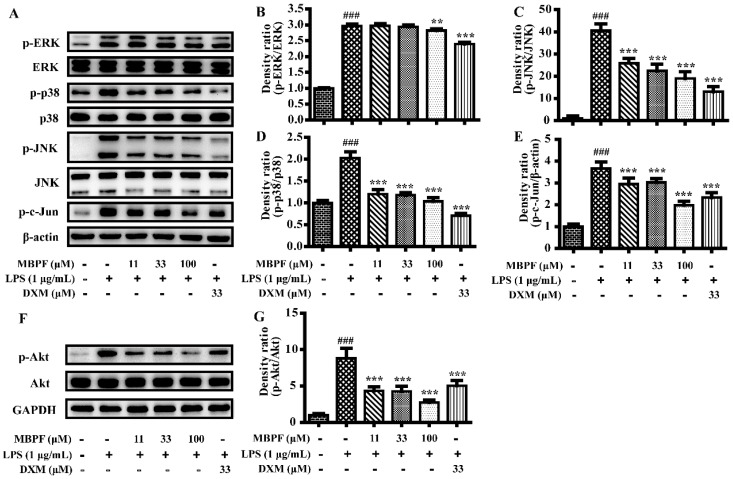
Effects of MBPF on MAPK and the PI3K/Akt signaling pathway in LPS-induced RAW 264.7 cells. The cells were pretreated with different concentrations of MBPF (0, 11, 33, and 100 μM) for 2 h and were then stimulated with or without LPS (1 μg/mL) for 30 min. The protein levels of phospho- extracellular regulated kinases (ERK1/2/ERK1/2), phospho-p38/p38, phospho-c-Jun N-terminal kinases (JNK/JNK), and phospho-c-Jun/β-actin were analyzed using Western blot (**A**–**E**). The p-Akt/Akt proteins were also determined using Western blot ((**F**) and (**G**)). Data represent the mean ± SE from three separate experiments. (^###^ *p* < 0.001 vs*.* control group; ** *p* < 0.01, *** *p* < 0.001 vs*.* LPS group).

The current study further investigated the molecular mechanisms of MBPF against LPS-induced inflammatory reaction in RAW 264.7 cells. It is known that MAPK, PI3K/Akt and NF-κB signaling pathways play important roles in the process of inflammation [17,18,19]. The MAPK pathway includes the extracellular regulated kinases (ERK1/2), p38 MAPKs, and the c-Jun N-terminal kinases (JNK), and inhibiting the activation of MAPKs down-regulates the expression of various inflammatory genes [20]. To examine whether MBPF inhibits the activation of MAPK pathways, Western blot analyses were performed in RAW 264.7 cells that were pretreated with MBPF. As the results showed (Figure 6A–E), MBPF inhibited the phosphorylation of p38, ERK, JNK, and c-jun. In addition, previous studies have indicated that LPS-stimulated activation of PI3K/Akt signaling was involved in the progression of inflammation [21,22]. As shown in Figure 6F,G, LPS dramatically enhanced the phosphorylation of the Akt protein in RAW264.7 cells, and Akt phosphorylation was clearly suppressed by MBPF.

**Figure 7:**
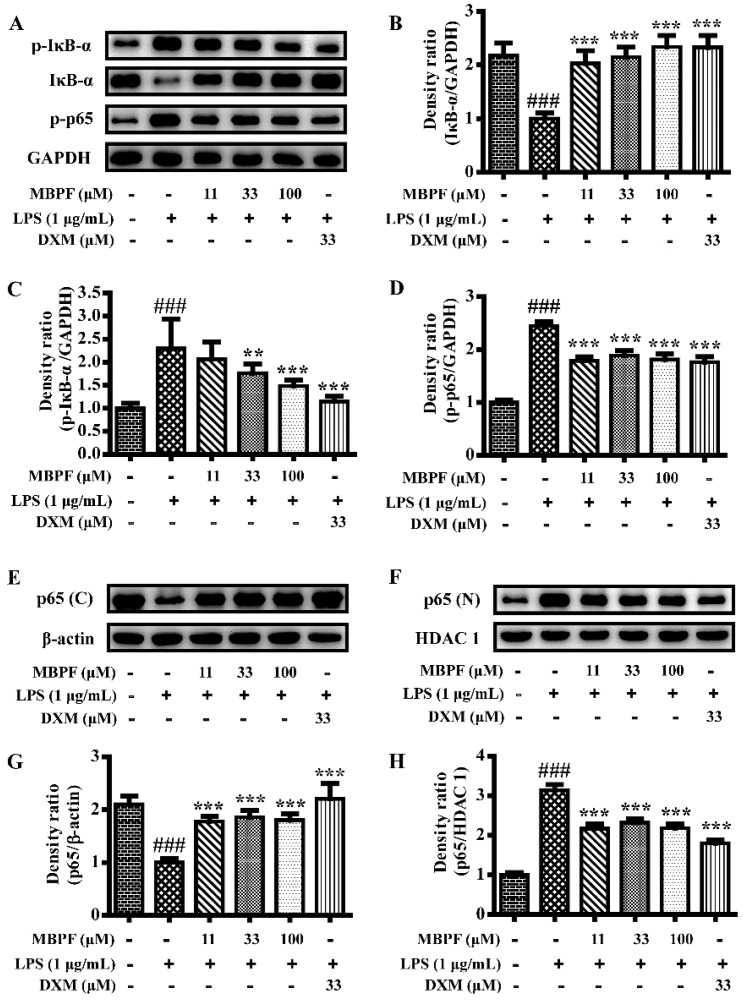
Effect of MBPF on LPS-induced activation of the nuclear factor κB (NF-κB) pathway in RAW264.7 cells. Cells were pretreated with various concentrations of MBPF (0, 11, 33, and 100 μM) for 2 h and were then stimulated with or without LPS (1 μg/mL) for 30 min. The protein levels of phospho-p65, phospho-IκB-α, and IκB-α were analyzed using Western blot (**A**–**D**). Cytosolic (C) and nuclear (N) protein were extracted to determine the levels of p65 using Western blot (**E**–**H**). The data represent the means ± S.E. of three independent experiments. (^###^ *p* < 0.001 vs*.* control group; ** *p* < 0.01, *** *p* < 0.001 vs*.* LPS group).

The NF-κB signaling pathway plays a crucial role in regulating the expression of inflammatory factors during an inflammatory response; as shown by the results of Western blot (Figure 7). The NF-κB signaling pathway was significantly activated by LPS in RAW 264.7 cells. However, MBPF distinctly inhibited the phosphorylation of p65, IκB-α, and attenuation of total IκB-α. Normally, NF-κB, in an inactive form, is associated with inhibitors of IκB that exist in the cytoplasm. When IκB-α was phosphorylated and degraded, the NF-κB p65 subunit was released and translocated to the nucleus to regulate gene transcription [23]. To clarify, we separated the cytosol and nucleus of the cells and determined the protein levels of p65. The Western blot results demonstrated that LPS increased the translocation of p65 to the nucleus, and MBPF clearly inhibited p65 translocation.

## 3. Experimental Section

### 3.1. Chemicals and Reagents

The monoterpenoids, PF, MPF, AF, PD, MBPF, BAF, PDA, GAF, and DAF, were isolated from *Paeonia lactiflora* Pall in our previously study. For the current study, they were dissolved in DMSO before use. Dulbecco’s Modified Eagle Medium (DMEM), fetal bovine serum (FBS), 0.25% trypsin, and penicillin-streptomycin-amphotericin (PSA) were purchased from Gibco BRL Co. Ltd. (Gaithersbug, MD, USA). The primary antibodies, such as anti-iNOS, anti-phospho-p38 (Thr180/Tyr182), anti-p38, anti-phospho-ERK1/2 (Thr202/Try204), anti-ERK, anti-phospho-JNK (Thr183/Tyr185), anti-JNK, anti-phospho-Akt (Ser473), anti-Akt, anti-phospho-c-Jun (Ser73), anti-phospho-p65 (Ser536), anti-p65, anti-p-IκBα (Ser32), IκBα anti-β-actin, anti-GAPDH, anti-HDAC 1, and secondary antibodies were purchased from Cell Signaling Technology (Danvers, MA, USA). Lipopolysaccharide (LPS) from *Escherichia coli* 055:B5, 3-[4,5-Dimethylthiazol-2-yl]-2,5-diphenyltetrazolium bromide (MTT), dexamethasone (DXM), and Trizol reagent were obtained from Sigma Chemical Co. (St. Louis, MO, USA). The Griess reagent, protein extraction kit, and BCA protein assay kit were obtained from the Beyotime Institute of Biotechnology (Beijing, China). Go Tag^®^ qPCR Master Mix and GoScript^TM^ Reverse Transcription System were purchased from Promega (Madison, WI, USA). Mouse TNF-α and the IL-6 ELISA kit were purchased from R&D systems (Abingdon, UK).

### 3.2. Cell Culture and Cytotoxic Assay

The RAW 264.7 cells were cultured in DMEM medium, supplemented with 10% heat-inactivated FBS, 2 mM l-glutamine, 100 U/mL penicillin, and 100 μg/mL streptomycin, at 37 °C under 5% CO_2_. The measurements of cell viability were from the MTT assay. RAW 264.7 cells were plated at 2 × 10^5^ cells per well in 96-well-plates. After incubation overnight, the cells were pretreated with compounds and DMSO for 2 h and then induced with LPS (1 μg/mL) for 24 h. After that, 20 μL of MTT (5 mg/mL) was added in order to incubate for 4 h at 37 °C under 5% CO_2_; then the cell medium was discarded and 150 μL of DMSO was added. After 5 min, the OD values of the culture plate at 570 nm was measured using a microplate reader.

### 3.3. Determination of Nitric Oxide (NO) Production

The cells were plated at 1 × 10^6^ cells/well in 6-well-plates and cultured overnight. The cells were pretreated with various compounds (33 μM) for 2 h and were then induced with LPS (1 μg/mL) for 24 h. After that, 50 μL of cell supernatant was collected into a new 96-well plate and 50 μL of Griess reagent I and 50 μL of Griess reagent II were added after incubation for 5 min at room temperature, the OD values of the 96-well plate were measured at 540 nm using a microplate reader. The production of nitrite was calculated using a standard curve of NaNO_2_.

### 3.4. Measurement of Cytokine (IL-6 and TNF-α)

The cells were seeded at the 1 × 10^6^ cells per well in 6-well-plates and cultured overnight. Then, the cells were, respectively, pretreated with various compounds (0, 11, 33, and 100 μM) and dexamethasone (DXM, 33 μM) for 2 h. After being stimulated with LPS (1 μg/mL) for 12 h, the production of IL-6 and TNF-α in the culture supernatant was measured using Elisa kits, according to the manufacturer’s protocols. The OD values at 450 nm were measured using a microplate reader.

### 3.5. Quantitative Real-Time Polymerase Chain Reaction (PCR) Analysis

Raw 264.7 cells were seeded at 1 × 10^6^ cells/well in 6-well-plates and cultured overnight, and then pretreated with MBPF (0–100 μM) and dexamethasone (33 μM) for 2 h, respectively. Following this, the cells were stimulated with LPS (1 μg/mL) for 6 h. Total RNA was extracted with Trizol reagent, according to the manufacturer’s protocols. Two micrograms of total RNA were reverse-transcribed to cDNA with a reverse transcription system, according to the manufacturer’s protocols. Relative mRNA levels of genes were quantified using SYBR green chemistry using a Bio-Rad CFX Connect Real-Time system (Hercules, CA, USA) with a set of specific primers: The forward and reverse primers for iNOS were 5′-TGGAGCGAGTTGTGGATTGTC-3′ and 5′-GGTCGTAATGTCCAGGAAGTAG-3′, respectively; the primers for GAPDH were 5′-TTGCGACTTCAACAGCAACTC-3′ and 5′-GGTCTGGGATGGAAATTGTG-3′.

### 3.6. Western Blot Analyses

The RAW264.7 cells were pretreated with various concentrations of MBPF (0–100 μM) and dexamethasone (33 μM), respectively, for 2 h and were induced with LPS (1 μg/mL) for 30 min (or 24 h for the iNOS protein). Total protein, as well as cytoplasmic and nuclear proteins were extracted using radioimmunoprecipitation assay (RIPA) buffer and the protein extraction kit (Beyotime, Beijing, China), according to the manufacturer’s instructions. The protein content was measured with a BCA protein assay kit (Beyotime, Beijing, China), protein samples (20–60 μg) were separated on 10% SDS-PAGE and transferred to 0.45 μm pore size polyvinylidene difluoride membranes (Millipore, Billerica, MA, USA). After being blocked with 5% skimmed milk for 1 h, the membranes were incubated with primary antibodies at 4 °C overnight, and then incubated with the conjugated secondary antibodies at room temperature for 1 h. Blots were exposed using the enhanced chemiluminescence detection kit (Millipore, Billerica, MA, USA). The protein signals were analyzed using Bio-Rad ChemiDoc™ XRS + System (Bio-Rad, Hercules, CA, USA).

### 3.7. Statistical Analyses

The data were expressed as the mean ± S.E. of at least three independent experiments. The statistical significance of the differences between the means were determined using the Student’s *t*-test. The *p* values < 0.05 (*) were considered significant, *p* values < 0.01 (**) and *p* < 0.001 (***) were considered highly significant.

## 4. Conclusions

In conclusion, most of the paeoniflorin, paeonidanin, and albiflorin derivatives from *P. lactiflora* possessed anti-inflammatory effects. Among them, paeoniflorins and paeonidanins presented stronger anti-inflammatory activities than albiflorin derivatives. Paeoniflorin derivative MBPF showed a stronger inhibition on the production of NO, IL-6, and TNF-α. The molecular mechanism investigations indicated that MBPF suppressed the production of pro-inflammatory cytokines via inhibition of the signaling pathways of MAPK, PI3K/Akt, and NF-κB in RAW 264.7 cells. These active monoterpenoids from *Radix Paeoniae Alba* may serve as potential leads for the development of anti-inflammatory agents.

## References

[1] Wang D., Tan Q.R., Zhang Z.J. (2013). Neuroprotective effects of paeoniflorin, but not the isomer albiflorin, are associated with the suppression of intracellular calcium and calcium/calmodulin protein kinase II in PC12 cells. J. Mol. Neurosci..

[2] Song W.H., Cheng Z.H., Chen D.F. (2013). Anticomplement monoterpenoid glucosides from the root bark of *Paeonia suffruticosa*. J. Nat. Prod..

[3] Jiang D., Chen Y., Hou X., Xu J., Mu X., Chen W. (2011). Influence of *Paeonia lactiflora* roots extract on cAMP-phosphodiesterase activity and related anti-inflammatory action. J. Ethnopharmacol..

[4] Duan W.J., Yang J.Y., Chen L.X., Zhang L.J., Jiang Z.H., Cai X.D., Zhang X., Qiu F. (2009). Monoterpenes from *Paeonia albiflora* and their inhibitory activity on nitric oxide production by lipopolysaccharide-activated microglia. J. Nat. Prod..

[5] Ding L., Zhao F., Chen L., Jiang Z., Liu Y., Li Z., Qiu F., Yao X. (2012). New monoterpene glycosides from *Paeonia suffruticosa* Andrews and their inhibition on NO production in LPS-induced RAW 264.7 cells. Bioorg. Med. Chem. Lett..

[6] Fu Q., Yu T., Yuan H.M., Song Y., Zou L. (2015). Paeonidanins F-H: Three new dimeric monoterpene glycosides from *Paeonia lactiflora* and their anti-inflammatory activity. Phytochem. Lett..

[7] He X., Han L., Huang X. (2011). A new phenolic glucoside from *Paeonia lactiflora*. Chin. Herbal Med..

[8] Zhao D.D., Jiang L.L., Li H.Y., Yan P.F., Zhang Y.L. (2016). Chemical Components and Pharmacological Activities of Terpene Natural Products from the Genus *Paeonia*. Molecules.

[9] Wang Q.S., Gao T., Cui Y.L., Gao L.N., Jiang H.L. (2014). Comparative studies of paeoniflorin and albiflorin from *Paeonia lactiflora* on anti-inflammatory activities. Pharm. Biol..

[10] Zhang J., Dou W., Zhang E., Sun A., Ding L., Wei X., Chou G., Mani S., Wang Z. (2014). *Paeoniflorin abrogates* DSS-induced colitis via a TLR4-dependent pathway. Am. J. Physiol.-Gastrointestinal Liver Physiol..

[11] Zhang C.S., Yang L., Zhang A.L., May B.H., Yu J.J., Guo X., Lu C., Xue C.C. (2016). Is oral Chinese herbal medicine beneficial for psoriasis vulgaris? A meta-analysis of comparisons with acitretin. J. Alterna. Complement. Med..

[12] Li K.K., Zhou X., Wong H.L., Ng C.F., Fu W.M., Leung P.C., Peng G., Ko C.H. (2016). In vivo and in vitro anti-inflammatory effects of Zao-Jiao-Ci (the spine of *Gleditsia sinensis* Lam.) aqueous extract and its mechanisms of action. J. Ethnopharmacol..

[13] Laflamme N., Rivest S. (2001). Toll-like receptor 4: the missing link of the cerebral innate immune response triggered by circulating gram-negative bacterial cell wall components. FASEB J..

[14] Jayasooriya R.G., Lee K.T., Lee H.J., Choi Y.H., Jeong J.W., Kim G.Y. (2014). Anti-inflammatory effects of beta-hydroxyisovalerylshikonin in BV2 microglia are mediated through suppression of the PI3K/Akt/NF-kB pathway and activation of the Nrf2/HO-1 pathway. Food Chem. Toxicol..

[15] Akira S., Takeda K. (2004). Toll-like receptor signalling. Nat. Rev. Immunol..

[16] Kim Y.H., Choi K.H., Park J.W., Kwon T.K. (2005). LY294002 inhibits LPS-induced NO production through a inhibition of NF-κB activation: Independent mechanism of phosphatidylinositol 3-kinase. Immunol. Lett..

[17] Cargnello M., Roux P.P. (2012). Activation and function of the MAPKs and their substrates, the MAPK-activated protein kinases. Microbiol. Mol. Biol. Rev..

[18] Thalhamer T., McGrath M., Harnett M. (2008). MAPKs and their relevance to arthritis and inflammation. Rheumatology.

[19] Yan T., Yu X., Sun X., Meng D., Jia J.M. (2016). A new steroidal saponin, furotrilliumoside from Trillium tschonoskii inhibits lipopolysaccharide-induced inflammation in Raw264. 7 cells by targeting PI3K/Akt, MARK and Nrf2/HO-1 pathways. Fitoterapia.

[20] Hossen M.J., Kim M.Y., Cho J.Y. (2016). MAPK/AP-1-Targeted anti-inflammatory activities of *Xanthium strumarium*. Am. J. Chin. Med..

[21] Xu X., Li H., Hou X., Li D., He S., Wan C., Yin P., Liu M., Liu F., Xu J. (2015). Punicalagin induces Nrf2/HO-1 expression via upregulation of PI3K/AKT pathway and inhibits LPS-induced oxidative stress in RAW264. 7 macrophages. Mediators Inflammation.

[22] Park C.M., Jin K.S., Lee Y.W., Song Y.S. (2011). Luteolin and chicoric acid synergistically inhibited inflammatory responses via inactivation of PI3K-Akt pathway and impairment of NF-κB translocation in LPS stimulated RAW 264.7 cells. Eur. J. Pharmacol..

[23] Fujioka S., Niu J., Schmidt C., Sclabas G.M., Peng B., Uwagawa T., Li Z., Evans D.B., Abbruzzese J.L., Chiao P.J. (2004). NF-κB and AP-1 connection: mechanism of NF-κB-dependent regulation of AP-1 activity. Mol. Cell. Biol..

